# Effects of web‐based interventions on quality of life among patients with breast cancer: A systematic review and meta‐analysis of randomized controlled trials

**DOI:** 10.1002/cam4.70230

**Published:** 2024-10-03

**Authors:** Lorinda A. Coombs, Myoungsuk Kim

**Affiliations:** ^1^ School of Nursing, Lineberger Cancer Institute University of North Carolina‐Chapel Hill Chapel Hill North Carolina USA; ^2^ College of Nursing Kangwon National University Chuncheon Republic of Korea

**Keywords:** breast cancer, internet‐based intervention, meta‐analysis, psycho‐oncology, psychosocial, quality of life

## Abstract

**Objective:**

Patients with breast cancer experience decreased quality of life due to various physical and psychological challenges. Web‐based interventions are accessible, cost‐effective, and convenient for improving their quality of life. This study evaluated whether web‐based interventions improve quality of life and included only randomized controlled trials (RCTs) with clear evidence.

**Methods:**

PubMed, Embase, Cochrane Library, CINAHL, Web of Science, and PsycINFO were searched for articles published until October 16, 2023. Inclusion criteria were RCTs evaluating the effect of web‐based interventions on quality of life in patients with breast cancer. The risk of bias was assessed with Cochrane's Risk of Bias Tool 2.0. Standardized mean differences were calculated with a random effects model using R version 4.0.3, and subgroup and moderator analyses were performed.

**Results:**

Since quality of life was measured using two different instruments in two studies, 21 comparisons were analyzed from 19 RCTs. As a result, the findings suggest that web‐based interventions have a small effect size on improving the quality of life for patients with breast cancer (SMD = 0.27, 95% confidence intervals [CIs]: 0.15–0.38, *p* = 0.03). Heterogeneity was found to be low (*I* ^2^ = 40%). The quality‐of‐life subdomain results showed a moderate effect size on the physical functioning and a small effect size on the cognitive and emotional functioning of patients with breast cancer but no significant impact on their role or social functioning.

**Conclusions:**

Web‐based interventions are effective in improving patients' quality of life with breast cancer; they also improve physical, cognitive, and emotional functioning. However, evidence regarding intervention methods remains inconclusive due to the limited number of RCTs, necessitating further research.

## 1 BACKGROUND

The most common cancer for women is breast cancer, which is the fifth cause of cancer‐related deaths globally.^1^ In 2020, the mortality‐to‐incidence ratio for breast cancer was 0.30 worldwide, 0.41 in Africa, 0.22 in the Americas, and the lowest in South Korea (0.12).^2^ In Europe, breast cancer mortality rates decreased by an annual average of 2%–4% between 1990 and 2017,^3^ and in the United States, there was a 43% decrease in mortality from 1975 to 2020.^4^ This decline in mortality is attributed to early detection and improved treatment methods, which have led to longer survival times among patients with breast cancer. As a result of improved survival, the quality of life of patients with breast cancer has become an increasingly important topic.

Patients with breast cancer may experience severe physical and psychological distress during diagnosis and treatment, which can significantly impair their quality of life. Anxiety, depression, pain, and post‐traumatic stress disorder are common psychological disorders as a result of their experience and can dramatically impair quality of life.^5^ In addition to immediate symptoms from the diagnosis and treatment, people diagnosed with breast cancer may experience fear of recurrence, cognitive impairment, changes in role and function, altered interpersonal relationships, changes in body image due to treatment side effects, and psychological difficulties related to the diagnosis.^5^, ^6^, ^7^ Body image, in particular, serves as a significant predictor of quality of life in patients with breast cancer.^8^ Various cancer treatments can negatively impact how patients with breast cancer perceive their bodies. Such negative body image can adversely affect not only the patient's emotional and psychological state but also have a major detrimental impact on their overall quality of life.^9^, ^10^ Furthermore, many physical issues, such as chronic pain, fatigue, and sleep disturbances, persist throughout treatment.^11^ Consequently, their quality of life is diminished, and their treatment adherence and outcomes are negatively impacted.^7^

Interventions are required to enhance the quality of life for patients with breast cancer. Various interventions address these issues; however, providing and receiving in‐person interventions over an extended period can be burdensome for patients, providers, and healthcare systems. In contrast, web‐based interventions are highly accessible, reduce patients' dependence on in‐person interventions, and can be provided to numerous patients, thus saving therapists' time.^12^ They are also cost‐effective and time‐efficient, and patients can access them anytime.^13^ Furthermore, during the COVID‐19 pandemic, web‐based interventions allowed patients with breast cancer to improve their health without exposing them to COVID‐19, leading to high patient satisfaction.^14^, ^15^

Several randomized controlled trials (RCTs) have reported that web‐based interventions may be effective in improving the quality of life of patients and survivors of breast cancer.^12^, ^16^, ^17^ However, others have suggested that web‐based interventions may not be effective in enhancing the quality of life of patients with breast cancer.^18^, ^19^ Still, other studies have reported that web‐based interventions may be an additional burden for cancer survivors^20^ and too time‐consuming for patients with breast cancer.^21^

Previous meta‐analyses have investigated the effects of web‐based interventions on the quality of life of patients with breast cancer. One meta‐analysis^22^ focused on broader aspects of eHealth, including mHealth, for patients with breast cancer. Some meta‐analyses examined the effects of web‐based self‐management interventions on the quality of life of patients with cancer.^23^, ^24^ However, they did not focus exclusively on patients with breast cancer and included both RCTs and non‐RCTs.^23^, ^24^ Moreover, the number of studies they analyzed was very small, making it difficult to obtain reliable evidence. We are unaware of any systematic review and meta‐analysis that has evaluated the effectiveness of web‐based interventions in improving the quality of life of patients with breast cancer by including only RCTs with clear evidence. Therefore, this study aimed to determine whether web‐based interventions help enhance the quality of life of patients with breast cancer by including only RCTs with clear evidence.

## 2 METHODS

Our systematic review and meta‐analysis followed the Preferred Reporting Items for Systematic Reviews and Meta‐analyses (PRISMA) guidelines for reporting (see Table S1).^25^ This review protocol was registered in the International Prospective Register of Systematic Reviews (PROSPERO) (CRD42023467420).

### 2.1 Information sources and search strategies

We comprehensively searched the PubMed, Embase, Cochrane Library, CINAHL, Web of Science, and PsycINFO databases from their inception to October 16, 2023. Search strategies were developed with MeSH (Medical Subject Headings), entry terms, Embase Subject Headings, and free‐text keywords. We used Boolean operators “AND” and “OR” to refine, and no language restrictions were imposed. The subject headings and keywords were breast cancer, web‐based interventions, and quality of life, and they were also updated in Tables S1–S6. The authors also conducted a manual search by reviewing the references in previous studies.

### 2.2 Eligibility criteria

Inclusion criteria were: (1) participants: adults ≥18 years who had received a diagnosis of breast cancer. We included all treatment modalities (e.g., surgery, adjuvant chemotherapy, and radiotherapy) and stage of cancer; (2) intervention: web‐based intervention using computer, internet‐based mobile phone, and tablet; (3) comparison: usual care, standard care, waiting list, standard care, and basic care (e.g., general information) provided identically to the intervention group; (4) outcomes: quality of life; (5) study design: randomized controlled trials (RCTs).

Studies were excluded under the following conditions: (1) studies that utilized an app, text message, or telephone call; (2) studies that combined a web‐based intervention with other interventions; (3) studies lacking sufficient statistical values for meta‐analysis; (4) Gray literature, including unpublished materials, research reports, policy documents, and government reports.

### 2.3 Study selection and data extraction

All the retrieved documents were managed using EndNote 20 reference software (Clarivate Analytics, Philadelphia, PA, USA). After removing duplicates, two reviewers (L.A.C. and M.K.) screened titles and abstracts independently. Next, both reviewers (L.A.C. and M.K.) independently reviewed the full‐text articles to assess eligibility. Disagreements at any stage were discussed until a consensus was reached.

After one reviewer (M.K.) extracted data from five studies, any data that needed to be added or removed from the extraction table was discussed with another author (L.A.C), and the table was finalized. Two reviewers (L.A.C. and M.K.) independently extracted data from 10 papers each and cross‐verified the extracted data. The extracted data included the first author, year of publication, country, study design, setting, participants, cancer stage, treatment category, sample size, mean age (standard deviation), intervention characteristics (type and duration), outcome measurements, control group, and completion rate. Disagreements related to data extraction were resolved through discussions.

### 2.4 Assessment of risk of bias

For the final selected studies, two review authors (L.A.C. and M.K.) independently evaluated the risk of bias using Cochrane's Risk of Bias Tool 2.0 for RCTs.^26^ Disagreement assessments were resolved through itinerant discussions until a consensus was reached. Risk of bias was assessed based on five domains: (1) randomization process, (2) deviations from intended interventions, (3) missing outcome data, (4) measurement of the outcome, and (5) selection of reported results. For each study, the overall risk of bias was classified as “low,” “some concerns,” or “high” based on the assessment of the five domains. The overall risk of bias was classified as “low” when all five domains were assessed as “low” and “some concerns” when no domain was evaluated as “high.” Still, one or more domains were assessed as “some concerns” and “high” when one or more domains were assessed as “high.” When there were differences in opinion, a third expert was included to reach a consensus.

### 2.5 Synthesis and statistical analysis

Statistical analysis was conducted using the R package Meta version 4.0.3 (R Foundation for Statistical Computing, Vienna, Austria). We calculated the effect size using standardized mean difference (SMD; Hedges' ĝ) with 95% confidence intervals (CIs) because different tools assessed quality of life. Moreover, we chose random effects models owing to variations in intervention methods, participants, and other factors across the studies.^27^ The overall pooled effect size was determined by calculating the weighted average of the effect sizes derived from all studies. Weights were assigned using the inverse of variance to determine the contribution of each study. Effect sizes were interpreted based on Cohen's criteria: (1) small (0.2 ≤ SMD <0.5), (2) moderate (0.5 ≤ SMD <0.8), or (3) large (SMD ≥0.8).^28^

Statistical heterogeneity among studies was assessed using *I* ^2^ statistics, with the following interpretations: 0%–40% indicating “might not be important,” 30%–60% suggesting “may represent moderate heterogeneity,” 50%–90% indicating “may represent substantial heterogeneity,” and 75%–100% indicating “considerable heterogeneity.”^29^ To investigate between‐study heterogeneity, we conducted subgroup analyses based on categorical variables such as study settings, intervention duration, measurement tool, and intervention method. Continuous moderators, such as sample size, mean age, and completion rate, were also examined using a meta‐regression analysis.

To evaluate publication bias, we utilized a funnel plot to show symmetry in the distribution of effect sizes. Next, we conducted Egger's regression test to confirm the presence of publication bias statistically. We then assessed the robustness of pooled estimates with a sensitivity analysis.

## 3 RESULTS

### 3.1 Study selection

**FIGURE 1:**
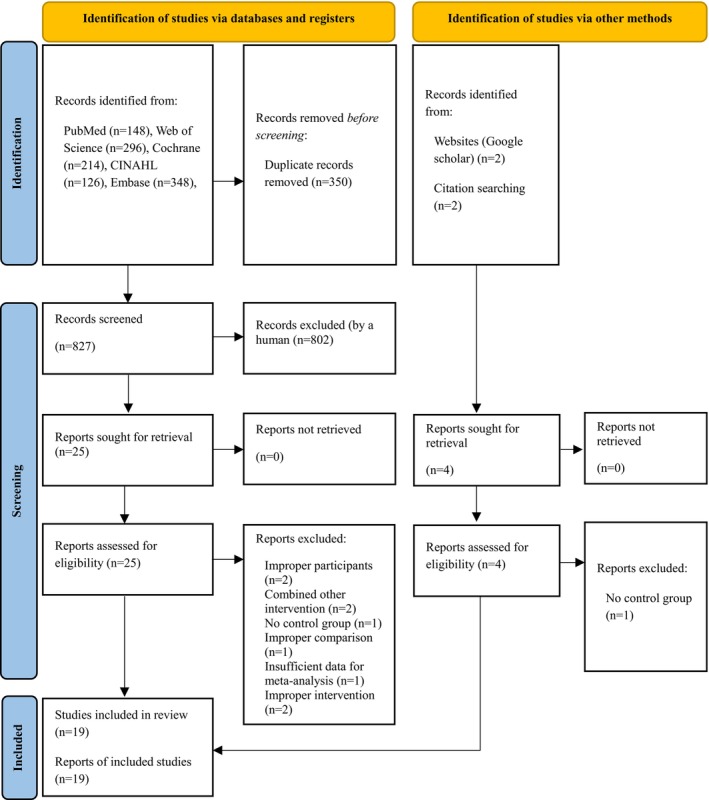
Flowchart summarizing the process of study selection.

A total of 1177 articles were retrieved from six databases. After removing 350 duplicates, 827 articles remained. We screened titles and abstracts to meet eligibility criteria, after which 802 articles were excluded. Subsequently, we reviewed the full text of the 25 remaining articles. From the 25, nine articles were excluded for a variety of reasons: (1) two did not meet the participant criteria,^30^, ^31^ (2) two combined web‐based interventions with other interventions,^32^, ^33^ (3) one lacked a control group,^34^ (4) one made improper comparisons between the intervention and control groups,^35^ (5) one had insufficient data for meta‐analysis,^36^ and (6) two did not meet the intervention criteria.^37^, ^38^ This left 16 articles for data analysis. Four more articles were identified from website searches and previous studies. One was excluded for the lack of a control group (*n* = 1).^39^ The final data analysis included 16 articles^12^, ^16^, ^17^, ^18^, ^19^, ^40^, ^41^, ^42^, ^43^, ^44^, ^45^, ^46^, ^47^, ^48^, ^49^, ^50^ from the 6 databases and 3 articles^51^, ^52^, ^53^ from manual searches (Figure 1).

### 3.2 Characteristics of the included studies

Table 1 summarizes the characteristics of the included studies. The publication years ranged from 2005 to 2023, with 73.68% of studies published after 2015. Studies were conducted in Europe (*n* = 10), the United States (*n* = 4), Asia (*n* = 3), Eurasia (*n* = 1), and Oceania (*n* = 1). Settings included hospitals (*n* = 11), community (*n* = 7), and combined community and hospital settings (*n* = 1).

**TABLE 1:** Characteristics of the randomized controlled trial studies (*N* = 19).

Author (year) country	Setting	Participants	Cancer stage	Sample size		Mean age (SD)	
				E	C	E	C
Abrahams (2017) Netherlands	8 hospitals	BC patients who received curative treatment within the last 3 months	I to III	61	64	52.5 (8.2)	50.5 (7.6)
Admiraal (2017) Netherlands	2 hospitals	BC patients who finished curative treatment within the last six months	I to III	61	59	53.1 (9.8)	53.2 (8.5)
Akkol‐Solakoglu (2023) Ireland or UK	Community	BC patients who finished primary cancer treatment	0 to 4	49	23	47.12 (7.92)	49.30 (9.66)
Atema (2019) Netherland	12 community and hospitals	BC patients who were diagnosed at the age of 50 or younger, and who had undergone chemotherapy and/or oophorectomy completed between 4 months and 5 years prior to study entry, except for trastuzumab use	NR	79	80	47.7 (5.73)	47.0 (5.50)
Changrani (2008) USA	Virtual community	Spanish‐dominant‐speaking immigrant women with BC	NR	42	20	46.2 (12.1)	50.8 (13.9)
Chee (2017) USA	Community	Asian American BC patients who have survived less than 5 years	NR	35	30	46.1 (10.6)	48.0 (11.1)
David (2011) German	Community	BC patients	I to IV	31	34	48.2 (9.2)	45.9 (7.8)
Feeman (2015) USA	Community	Breast cancer survivors who are at least 6 weeks out from completing cancer treatment.	0 to IV	19	43	55.57 (9.88)	55.28 (7.90)
Galiano‐Castillo (2016) Spain	1 hospital	BC patients who completed adjuvant therapy except hormone treatment	I to IIIA	39	36	47.4 (9.6)	49.2 (7.9)
Holtdirk (2021) Germany	Community	BC patients diagnosed within 5 years who have completed treatment	NR	141	165	50.07 (8.51)	49.8 (7.98)
Hummel (2017) Netherlands	10 hospitals	BC patients diagnosed between 6 months and 5 years before study entry, who have completed treatment (excluding maintenance endocrine therapy or immunotherapy)	NR	69	82	51.1 (7.2)	51.5 (7.7)
Korkmaz (2020) Turkey	2 hospitals	BC patients who were hospitalized and underwent breast surgery, including modified radical mastectomy or breast‐conserving surgery	NR	22	22	44.63 (7.53)	49.95 (8.28)
Lee (2014) Korea	3 hospitals	BC patients who underwent curative surgery and completed cancer treatment 1 year prior to study	0 to III	29	28	41.5 (6.3)	43.2 (5.1)
Owen (2005) USA	1 hospital	Early‐stage breast cancer	I to III	26	27	52.5 (8.6)	51.3 (10.5)
Peng (2022) China	1 hospital	BC patients who have completed all treatments, except for hormonal therapy or Herceptin, and have no recurrence or metastasis	I to IV	28	29	NR	NR
Ryhänen (2013) Finland	1 hospital	Newly diagnosed BC patients	I to IV	47	43	54.4	55.7
van den Berg (2015) Netherlands	6 hospitals	BC patients who completed curative‐intent primary treatment 2 to 4 months before the baseline assessment	NR	63	72	51.44 (8.30)	50.18 (9.15)
Wang (2022) China	1 hospital	BC patients who have completed surgery 1 to 24 months ago	0 to IV	51	52	45.37 (7.59)	48.17 (8.05)
White (2018) Australia	Community	BC survivors who are young and are approximately 6 months post‐diagnosis	I to II	171	165	43.6 (5.0)	43.9 (5.3)

Abbreviations: BC, breast cancer; BCS, breast cancer survivors; C, control group; E, experimental group; NR, not reported; SD, standard deviation.

The cancer stage varied across the studies and included stages from 0 to IV; however, seven studies did not report this information. The intervention groups' sample sizes ranged from 19 to 171 individuals, whereas the control group samples ranged from 20 to 165. The mean age of the study participants ranged from 41.5 to 55.57, and one study did not report the mean age (Table 1).

### 3.3 Characteristics of interventions

The web‐based interventions included a variety of content, encompassing psychosocial interventions, such as mindfulness and cognitive behavioral therapy (*n* = 14), self‐management interventions, such as educative information and exercise programs (*n* = 5), and support groups (*n* = 2). The duration of the interventions ranged from 1 to 9 months. Although measurement tools varied, the EORTC Core Quality of Life questionnaire (EORTC‐QLQ‐C30) (*n* = 8) was used predominantly, followed by the Functional Assessment of Cancer Therapy‐Breast (FACT‐B) (*n* = 6). The control group primarily received usual care (*n* = 10), followed by a waiting list (*n* = 4). The completion rate, the proportion of individuals included in the final analysis out of all participants initially randomized, varied from 48.87% to 100% (Table 2).

**TABLE 2:** Characteristics of intervention of the included studies (*N* = 19).

Author (year)	Intervention	Type of intervention	Duration of intervention	Outcome measurements	Control group	Completion rate
Abrahams (2017)	iCBT (internet‐based CBT)	Psychosocial intervention	6 months	EORTC‐QLQ‐C30	Usual care	94.70
Admiraal (2017)	ENCOURAGE program (web‐based tailored psychoeducational program)	Psychosocial intervention	12 weeks	EORTC‐QLQ‐C30	Standard care	86.33
Akkol‐Solakoglu (2023)	iCBT (internet‐delivered CBT)	Psychosocial intervention	8 weeks	EORTC‐QLQ‐C30	Usual care	79.17
Atema (2019)	Self‐managed iCBT (internet‐based CBT)	Psychosocial intervention	6 weeks	SF‐36	Waiting list	94.08
Changrani (2008)	OSGs (online support groups) for immigrants with cancer	Support groups	30 weeks	FACT‐B	Usual care	91.18
Chee (2017)	∙ ICSG‐AA (internet cancer support groups for Asian American BCS) ∙ Internet resources related to Asian Americans' daily life	Support groups	1 month	FACT‐B	Internet resources related to Asian Americans' daily life	100.00
David (2011)	Psychosocial counseling via web‐based e‐mail	Psychosocial intervention	2 months	EORTC‐QLQ‐C30	Waiting list	48.87
Freeman (2015)	Telemedicine delivery (videoconferencing): telemedicine delivered imagery‐based behavioral intervention	Psychosocial intervention	4 weeks	∙ SF‐36 ∙ FACT‐B	Waiting list	90.00
Galiano‐Castillo (2016)	e‐CUIDATE system (Online system that facilities the development of remote rehabilitation): tailored exercise program	Self‐management	8 weeks	EORTC‐QLQ‐C30	Usual care	93.83
Holtdirk (2021)	∙ Optimune (a new CBT‐based Internet intervention) ∙ Usual care	Psychosocial intervention	3 months	WHOQOLBREF	Usual care	82.04
Hummel (2017)	Internet‐based CBT	Psychosocial intervention	24 weeks	SF‐36	Waiting list	89.35
Korkmaz (2020)	Web‐based education associated with the pre‐operative and post‐operative periods	Self‐management	1 month	SF‐36	Routine education	91.67
Lee (2014)	WSEDI (web‐based self‐management exercise and diet intervention program)	Self‐management	12 weeks	EORTC‐QLQ‐C30	Basic care (Booklet)	96.61
Owen (2005)	Self‐guided internet coping‐skills training exercise	Psychosocial intervention	12 weeks	∙ FACT‐B ∙ EQ‐5D	Waiting list	85.48
Peng (2022)	Online MBI (mindfulness‐based intervention)	Psychosocial intervention	6 weeks	EORTC‐QLQ‐C30	Usual care	95.00
Ryhänen (2013)	∙ BCPP (BC patient pathway) program: internet‐based patient educational program on BC patients' empowerment. ∙ Standard practice	Self‐management	9 months	Quality of life instrument ‐ Breast cancer patient version	Standard practice	91.84
van den Berg (2015)	∙ BREATH (BC e‐health): web‐based self‐management intervention to support the psychological adjustment of women after primary treatment. ∙ Usual care	Psychosocial intervention	16 weeks	EORTC‐QLQ‐C30	Usual care	90.00
Wang (2022)	iMBCR (internet‐delivered mindfulness‐based cancer recovery)	Psychosocial intervention	4 weeks	FACT‐B	Usual care	100.00
White (2018)	“*informe*” (information for me) website: self‐directed information resource	Self‐management	3 months	FACT‐B	Usual care	88.65

Abbreviations: BC, brest cancer; BCS, breast cancer survivors; CBT, cognitive behavioral therapy; EORTC QLQ‐C30, European Organization for Research and Treatment of Cancer Quality of Life Questionnaire C30; EQ‐5D, EuroQol–5D; FACT‐B, Functional Assessment of Cancer Therapy‐Breast; SF‐36, Short Form 36‐Item Health Survey; WHOQOL‐BREF, World Health Organization Quality of Life.

### 3.4 Risk of bias

**FIGURE 2:**
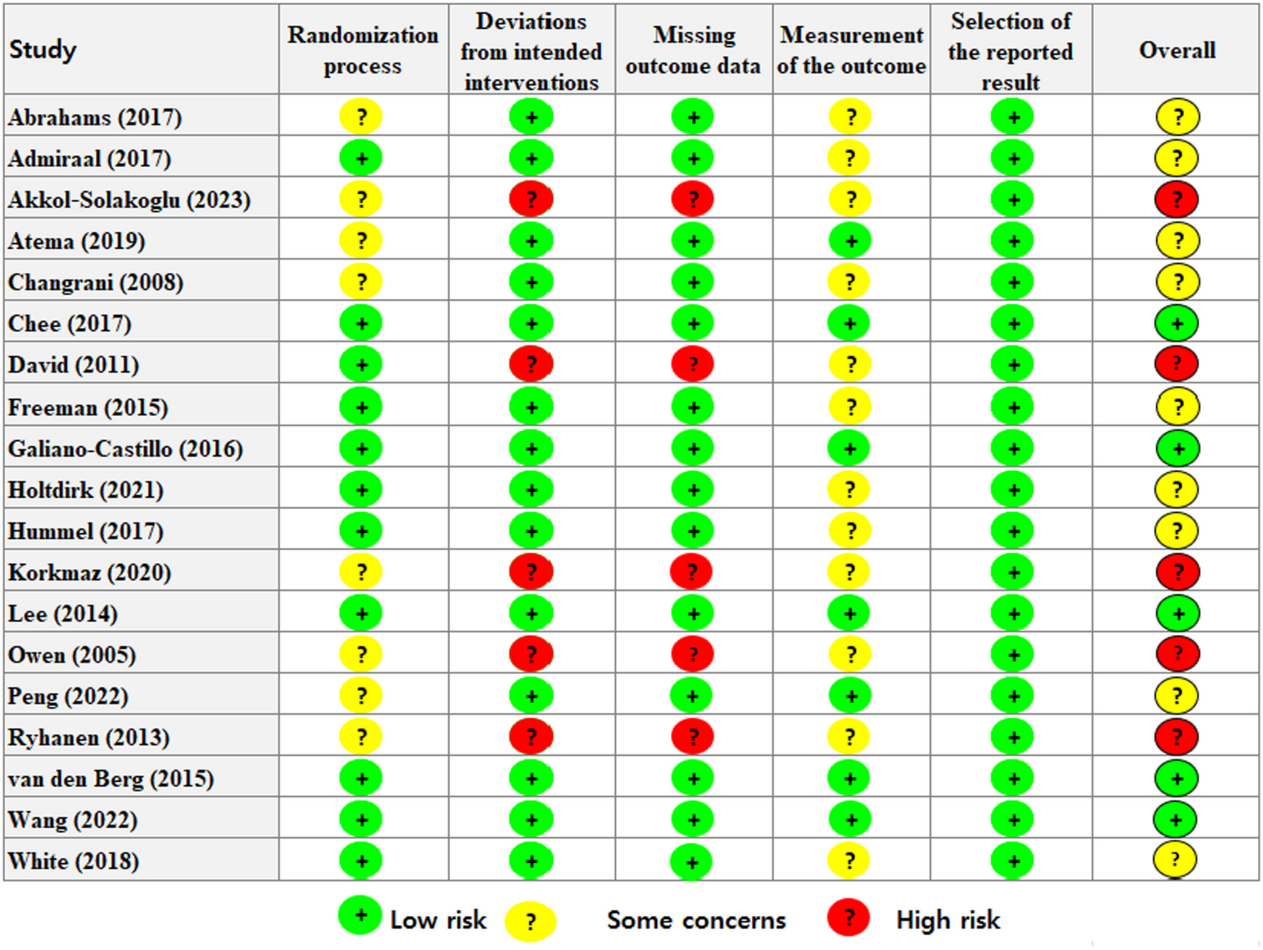
Risk‐of‐bias summary according to the revised Cochrane risk‐of‐bias 2.0 tool for randomized trials.

Risk of bias assessment results (Figure 2) for the 19 studies included. Five studies were assessed as “low,” and nine were assessed as “some concerns” due to inappropriate randomization processes and outcome measurements. Regarding the randomization process, concealment of the allocation sequence was inappropriate until participants were enrolled and assigned to the intervention group. Regarding outcome measurement, since the data was self‐reported by the participants, there were “some concerns” that the outcome assessment might have been influenced by knowledge of the intervention received. In five studies, the participants and those delivering the intervention were aware of the intervention group during the trial, and an appropriate analysis was not performed to estimate the intervention's effect. Moreover, the analysis of unanalyzed outcomes from randomly assigned groups substantially impacted the results. Additionally, in measuring the outcome, the risk of bias was assessed as “high” due to the potential for bias from missing data.

### 3.5 Effects of web‐based interventions on the quality of life of patients with breast cancer

**FIGURE 3:**
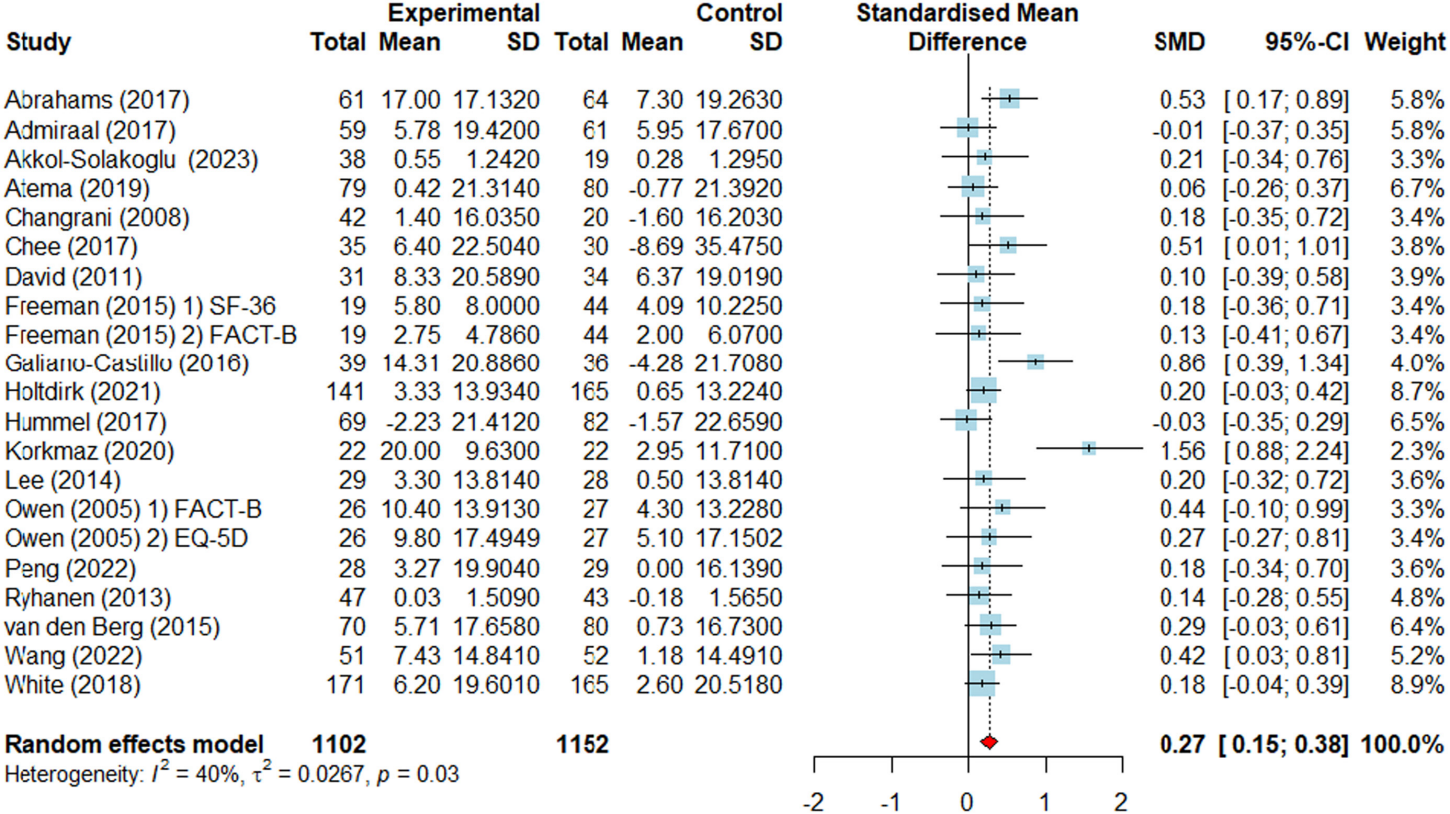
Forest plots: Effect of web‐based interventions on quality of life. CI, confidence interval; SMD, standardized mean difference.

We conducted a meta‐analysis of 21 RCTs from 19 studies since quality of life was measured using two different instruments in two of the studies. The pooled overall effect of web‐based interventions on the quality of life in patients with breast cancer showed a small effect size (SMD = 0.27, 95% CI = 0.15–0.38, *p* = 0.03). Heterogeneity testing showed *I* ^2^ = 40%, indicating a low level of heterogeneity (*p* < 0.03) (Figure 3).

### 3.6 Effects of web‐based interventions on functional domains of the quality of life of patients with breast cancer

**FIGURE 4:**
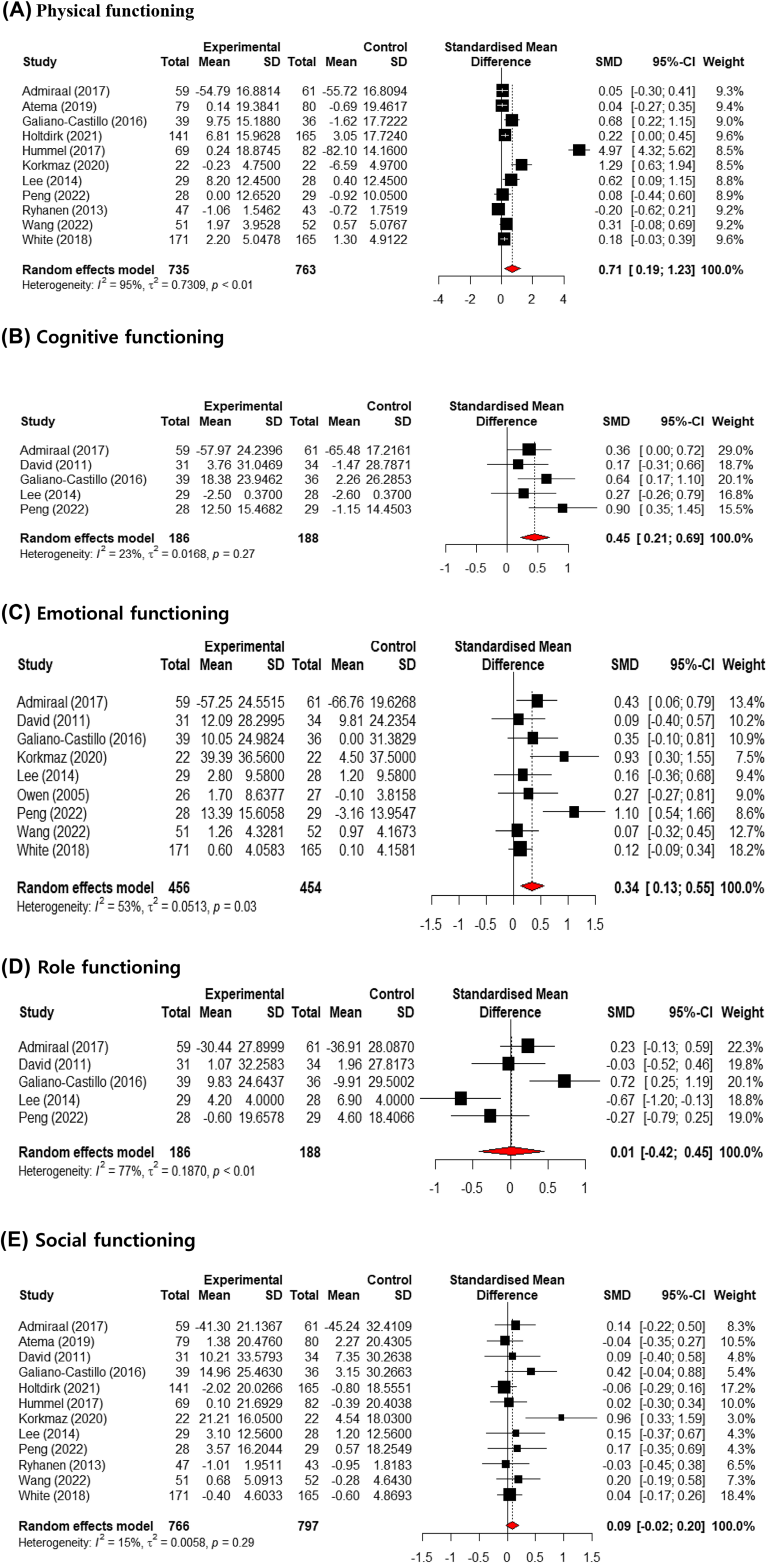
Forest plot for the effect of web‐based interventions on five functional domains. (A) physical, (B) cognitive, (C) emotional, (D) role, (E) social. CI, confidence interval; SMD, standardized mean difference.

We evaluated the effects of web‐based interventions on the physical, cognitive, emotional, role, and social domains of the quality of life of patients with breast cancer. Compared to the control group, web‐based interventions showed a moderate effect size on physical functioning (SMD = 0.71, 95% CI = 0.19–1.23, *p* < 0.01, *I* ^2^ = 95%). They also showed a small effect size on cognitive (SMD = 0.45, 95% CI = 0.21–0.69, *p* = 0.27, *I* ^2^ = 23%) and emotional functioning (SMD = 0.34, 95% CI = 0.13–0.55, *p* = 0.03, *I* ^2^ = 53%). However, no significant effect was observed on role and social functioning compared to the control group (Figure 4).

### 3.7 Subgroup and moderator analyses

We conducted moderator analyses to examine how specific variables operate and ascertain the differences in effects among subgroups. Our subgroup analyses showed web‐based interventions to be more effective when the study is conducted in a hospital setting (vs. a community setting) (SMD = 0.30, 95% CI = 0.15–0.45, *I* ^2^ = 61.1%), the intervention lasts less than 3 months (vs. 3 or more months) (SMD = 0.30, 95% CI = 0.14–0.47, *I* ^2^ = 60.0%), cancer‐specific measurement tools are used (vs, breast‐cancer‐specific, health‐related, and general tools) (SMD = 0.29, 95% CI = 0.08–0.50), and self‐management interventions are employed (vs. psychosocial interventions and support groups) (SMD = 0.43, 95% CI = 0.18–0.67) (Table 3). A meta‐regression analysis was conducted for continuous moderator variables, and this analysis revealed that the effect size of web‐based interventions is not significantly influenced by participants' mean age, completion rate, or sample size.

**TABLE 3:** Subgroup and moderator analysis.

Moderators	Subgroup	*k*	SMD	95% CI	*I* ^2^ (%)	Between groups	
						*Q*_*b*_ (df)	*p*_*b*_
Categorical moderators	Setting						
	Community	8	0.20	0.01, 0.39	0.0		
	Hospital	13	0.30	0.15, 0.45	61.1	0.67 (1)	0.411
	Duration of intervention						
	<3 months	12	0.30	0.14, 0.47	60.0	0.48 (1)	0.486
	≥3 months	9	0.22	0.05, 0.39	0.0		
	Measurement of QOL						
	Breast cancer‐specific (FACT‐B)	7	0.27	0.05, 0.49	0.0	0.16 (3)	0.983
	Cancer‐specific (EORTC‐QLQ‐C30)	8	0.29	0.08, 0.50	36.2		
	Health‐related (SF‐36, EQ‐5D)	5	0.26	−0.01, 0.53	77.9		
	General (WHOQOLBREF)	1	0.19	−0.26, 0.65	‐		
	Method of intervention						
	Psychosocial intervention	14	0.20	0.60, 0.34	0.0	2.71 (2)	0.258
	Self‐management	5	0.43	0.18, 0.67	80.4		
	Support group	2	0.35	−0.07, 0.79	0.0		

Continuous moderators	Variables	*k*	QM	*β*	SE	*p*	*R* ^2^ (%)
	Mean age	20	0.60	−0.014	0.018	0.436	0.0
	Completion rate	21	1.47	0.007	0.006	0.224	4.76
	Sample size	21	1.45	−0.001	0.001	0.227	0.0

Abbrevaitions: CI, confidence interval; df, degree of freedom; P_b_, P between groups; Q_b_, Q between groups; QM, Q moderator.

### 3.8 Publication bias

**FIGURE 5:**
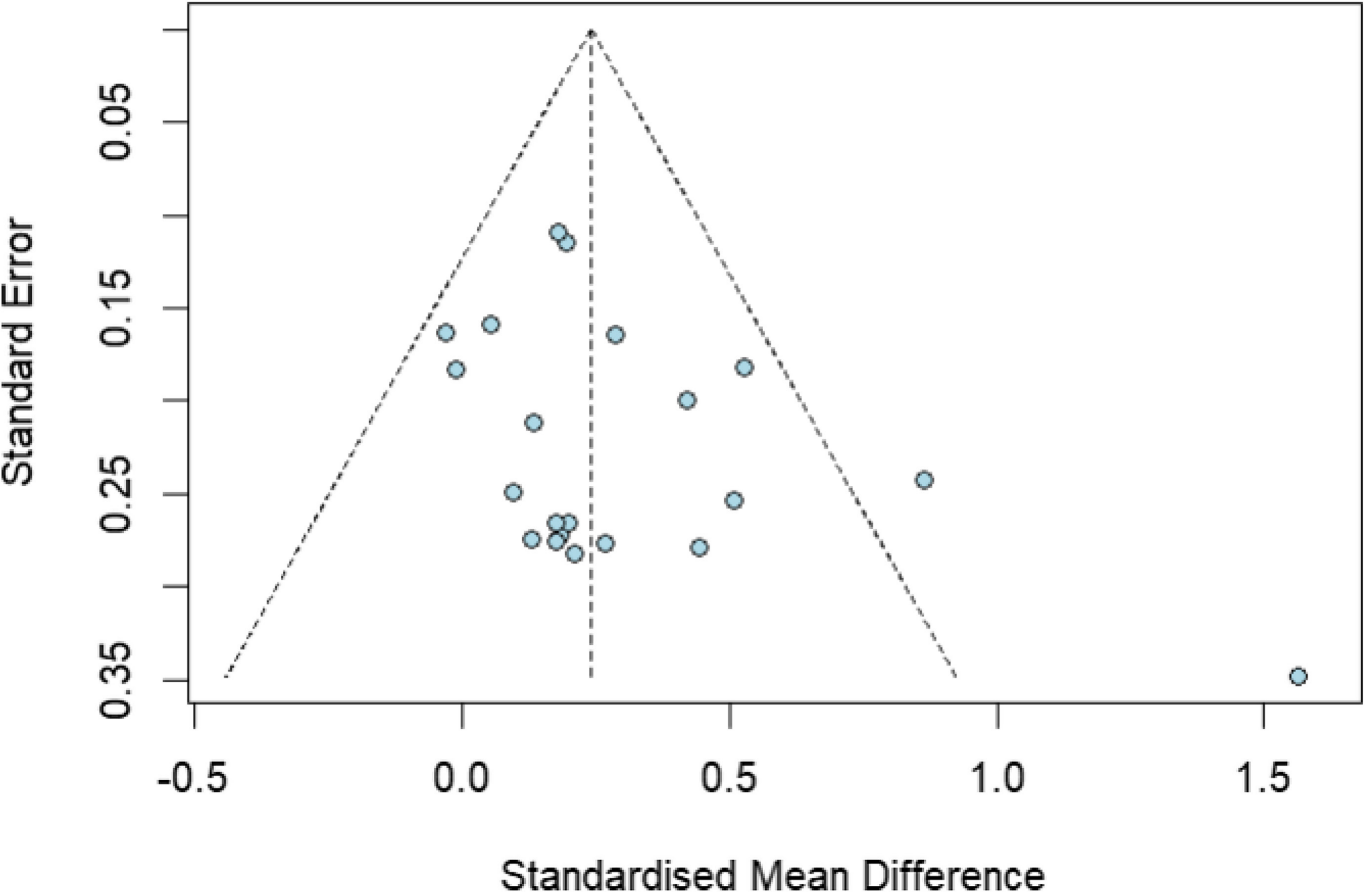
Forest plot of standard error by effect size for quality of life.

The assessment of publication bias began with a visual inspection of the funnel plot to determine its symmetry (Figure 5). Then, Egger's regression analysis was performed, and it confirmed the absence of a statistically significant asymmetry (*t* = 1.866, *p* = 0.077). This indicated the absence of publication bias.

### 3.9 Sensitivity analysis

**FIGURE 6:**
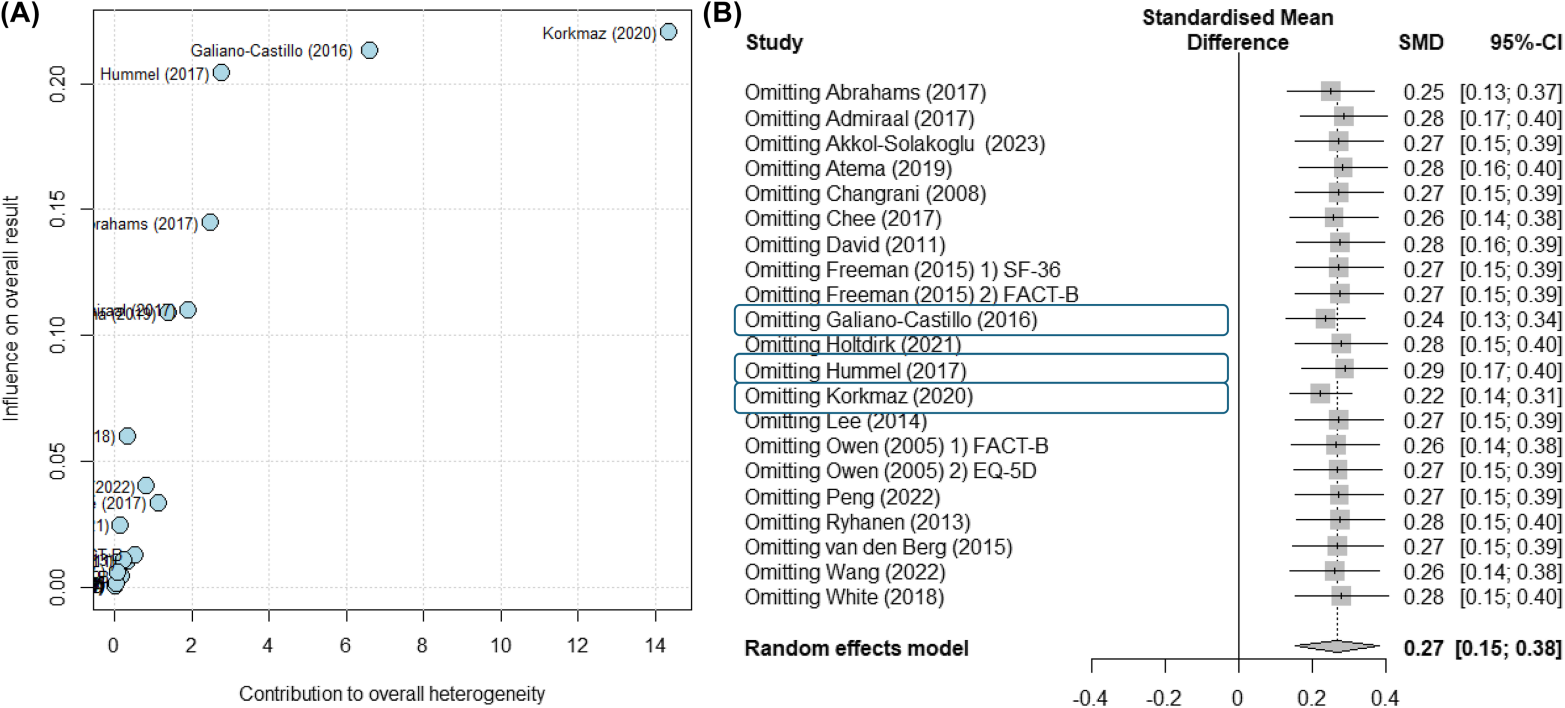
(A) Baujat plot. (B). Forest plot of sensitivity analysis. CI, confidence interval; SMD, standardized mean difference.

We identified the studies with the most significant influence on the overall effect size using the Baujat plot. Three studies (Korkmaz, Iyigun, Tastan^45^ Galiano‐Castillo et al.^17^ and Hummel et al.)^18^ were found to have the greatest influence (Figure 6A). When each study was removed individually, it did not significantly impact the overall effect size, demonstrating its reliability (Figure 6B). Our sensitivity analysis confirmed the validity of the findings regarding the effectiveness of web‐based interventions on the quality of life of patients with breast cancer.

## 4 DISCUSSION

This systematic review and meta‐analysis analyzed only robust RCTs and demonstrated the effectiveness of web‐based interventions in enhancing the quality of life of patients with breast cancer. It is particularly salient because it is the first study to confirm the distinct impact of web‐based interventions on the quality of life of individuals with breast cancer. Moreover, it included a substantial number of studies with low heterogeneity and no signs of publication bias; the findings demonstrate that web‐based interventions are a valuable approach for enhancing the quality of life of patients with breast cancer.

Our findings are somewhat similar to those of a previous study^22^ that found a small effect size of eHealth interventions in improving the quality of life of patients with breast cancer. However, that study differed from this one in terms of its comprehensive approach; it included mHealth and did not specifically focus on web‐based interventions. Our findings are also similar to those of another meta‐analysis.^54^ However, it did not specifically target patients with breast cancer, which found a small effect size of mHealth interventions in improving the quality of life of patients with cancer. Based on previous studies and our research, we can cautiously conclude that digital health interventions such as e‐Health, mHealth, and web‐based interventions may have a small effect size on improving the quality of life for cancer patients.

Our findings differ from those of a previous meta‐analysis^23^, ^24^ that found a large effect size of web‐based self‐management interventions in improving the quality of life of patients with cancer. However, those meta‐analyses included only self‐management interventions and did not incorporate other web‐based interventions. They also included non‐RCTs and a limited number of studies, including results with limited reliability. Web‐based interventions are convenient, accessible, and readily available to many patients.^12^, ^13^ They can be accessed through various operating systems and devices with an Internet connection (such as computers, tablets, and phones); they can be used without a mobile phone, and one need not install a mobile app to use them. In contrast, mobile app use requires a smartphone, must be installed and updated regularly, and different applications must be developed for other operating systems. Therefore, web‐based interventions may be more valuable and accessible than mHealth apps.

This review confirmed the impact of web‐based interventions on the quality of life for patients with breast cancer, with moderate effects on physical functioning and a small effect on cognitive and emotional functioning. A previous meta‐analysis examined the impact of eHealth but did not find a significant effect on any of the functional domains of the quality of life of patients with breast cancer.^19^ However, this study included web‐based interventions and mHealth, unlike our study, which focused solely on web‐based interventions. This shows that employing web‐based interventions can be a practical approach to enhancing the physical, cognitive, and emotional functioning of patients with breast cancer. Patients with breast cancer often experience physical difficulties, such as chronic pain, sleep disorders, and fatigue,^11^ and psychological and emotional challenges, including anxiety, depression, and cognitive impairment, during their cancer treatment.^5^ Web‐based interventions may serve as convenient and easily accessible aids in improving the physiological, cognitive, and emotional functioning of patients with breast cancer.

In our subgroup analyses, web‐based interventions exhibited a small effect size. The effects of web‐based interventions on improving the quality of life, according to the setting and duration of the intervention, were consistently observed to have a small effect size across all groups. In contrast, they exhibited a small effect size for breast cancer‐ and cancer‐specific measurement tools but no significant effect for health‐related and general measurement tools. No statistically significant differences were found between the groups, and a limited number of studies utilized health‐related and general measurement tools, suggesting the necessity for further research. However, these findings can be considered when formulating web‐based intervention strategies.

In our subgroup analyses based on the intervention method, web‐based interventions exhibited a small effect size for psychosocial and self‐management interventions but no significant effect for support groups. Our findings align with a previous study^24^ in which web‐based self‐management interventions were provided not only to patients with breast cancer but also to all patients, and a small size effect was found. However, they differ from a meta‐analysis^23^ that found a large effect size. This discrepancy may be because previous studies did not focus exclusively on RCTs and did not solely target patients with breast cancer. In contrast, this review provides more robust results, exclusively focusing on RCTs. Nevertheless, further research is warranted, given the limited number of studies included in the web‐based self‐management intervention subgroup and the lack of statistically significant differences between groups.

### 4.1 Study limitations

This review had three identifiable limitations. First, it included studies from various countries but not all nations, which limits the generalizability of its results to the entire global population. Second, this study included only published studies and excluded a variety of studies, such as gray literature, which can restrict the generalizability of its results. Third, in our subgroup analyses, both measurement tool and intervention method subgroups exhibited uncertain results owing to a limited number of studies and considerable heterogeneity. Therefore, interpreting these results should be cautiously approached, and further research is needed to enhance the robustness of our findings.

### 4.2 Clinical implications and directions for future research

This review exclusively included RCTs and secured enough studies to substantiate the utility of web‐based interventions in enhancing the quality of life of patients with breast cancer. These patients often experience prolonged physical and psychosocial distress during and after treatment, which significantly deteriorates their quality of life. Web‐based interventions serve as an accessible and convenient aid for numerous patients. Based on the results of our review, the development of web‐based interventions is warranted to enhance the quality of life of patients with breast cancer. The results also highlight the utility of web‐based interventions in improving such patients' physical, cognitive, and emotional functioning. Overall, our findings underscore the importance of developing and implementing web‐based interventions to enhance the overall quality of life of patients with breast cancer.

This review included studies predominantly conducted in Europe. Incorporating studies from other regions and countries with high incidence rates of breast cancer can strengthen evidence of the effectiveness of web‐based interventions in improving the quality of life of patients with breast cancer. Additionally, our analyses revealed a need for more research on interventions other than psychosocial interventions. Further research is required to determine the effects of web‐based interventions based on the intervention method.

## 5 CONCLUSION

This study shows that web‐based interventions are effective in improving the quality of life for patients with breast cancer. Additionally, its results confirm that web‐based interventions can enhance various aspects of the quality of life of such patients, particularly their physical, cognitive, and emotional functioning. However, due to the limited number of studies in the subgroup analyses, the evidence regarding specific methods of web‐based interventions is less reliable, indicating the need for further research. Our findings can contribute to the development of tailored intervention strategies to improve the quality of life for patients with breast cancer.

## AUTHOR CONTRIBUTIONS

**Lorinda A. Coombs:** Conceptualization (equal); data curation (equal); methodology (equal); validation (equal); writing – original draft (equal); writing – review and editing (equal). **Myoungsuk Kim:** Conceptualization (equal); data curation (equal); software (equal); supervision (equal); visualization (equal); writing – original draft (equal); writing – review and editing (equal).

## CONFLICT OF INTEREST STATEMENT

The authors declare that they have no conflicts of interest.

## Supporting information


File S1.

Table S1.
Table S2.
Table S3.
Table S4.
Table S5.
Table S6.

## Data Availability

The data supporting this study's findings are available upon reasonable request.
