# Supplementary material for: Effects of web‐based interventions on quality of life among patients with breast cancer: A systematic review and meta‐analysis of randomized controlled trials
Source: Cancer Med. 2024 Oct 3;13(18):e70230. doi: 10.1002/cam4.70230 (PMC11447276; doi:10.1002/cam4.70230)
Supplement: Supplementary file 2 — Table S1. Table S2. Table S3. Table S4. Table S5. Table S6. [file CAM4-13-e70230-s002.docx]

**Supplementary material**

**Supplementary** **Table 1. PubMed**

| Search # | MeSH Terms and Key Words | Articles Revealed |
| --- | --- | --- |
| #1 | "Breast neoplasms"[MeSH Terms] OR "Breast Neoplasm"[All Fields] OR "Breast Tumors"[All Fields] OR "Breast Tumor"[All Fields] OR "Breast Cancer"[All Fields] OR "Mammary Cancer"[All Fields] OR "Mammary Cancers"[All Fields] OR "Malignant Neoplasm of Breast"[All Fields] OR "Breast Malignant Neoplasm"[All Fields] OR "Breast Malignant Neoplasms"[All Fields] OR "Malignant Tumor of Breast"[All Fields] OR "Breast Malignant Tumor"[All Fields] OR "Breast Malignant Tumors"[All Fields] OR "Cancer of Breast"[All Fields] OR "Cancer of the Breast"[All Fields] OR "Human Mammary Carcinomas"[All Fields] OR "Human Mammary Carcinoma"[All Fields] OR "Human Mammary Neoplasms"[All Fields] | [457,380](https://pubmed-ncbi-nlm-nih-gov-ssl.libproxy.snu.ac.kr/?term=%28%22Breast+Neoplasm%22+OR+%22Breast+Tumors%22+OR+%22Breast+Tumor%22+OR+%22Breast+Cancer%22+OR+%22Mammary+Cancer%22+OR+%22Mammary+Cancers%22+OR+%22Malignant+Neoplasm+of+Breast%22+OR+%22Breast+Malignant+Neoplasm%22+OR+%22Breast+Malignant+Neoplasms%22+OR+%22Malignant+Tumor+of+Breast%22+OR+%22Breast+Malignant+Tumor%22+OR+%22Breast+Malignant+Tumors%22+OR+%22Cancer+of+Breast%22+OR+%22Cancer+of+the+Breast%22+OR+%22Human+Mammary+Carcinomas%22+OR+%22Human+Mammary+Carcinoma%22+OR+%22Human+Mammary+Neoplasms%22%29+OR+%28breast+cancer%5BMeSH+Terms%5D%29&sort=) |
| #2 | "Internet"[MeSH Terms] OR "Internet use"[MeSH Terms] OR "Internet Based Intervention"[MeSH Terms] OR "telemedicine"[MeSH Terms] OR "Internet Uses"[All Fields] OR "Web Usage"[All Fields] OR "Web Use"[All Fields] OR "Internet Usage"[All Fields] OR "Internet Based Intervention"[All Fields] OR "Internet-Based Interventions"[All Fields] OR "Web based intervention"[All Fields] OR "web based intervention"[All Fields] OR "Web-based Interventions"[All Fields] OR "Online Intervention"[All Fields] OR "Online Interventions"[All Fields] OR "Internet Intervention"[All Fields] OR "Internet Interventions"[All Fields] OR "Tele-Referral"[All Fields] OR "Tele-Referrals"[All Fields] OR "Virtual Medicine"[All Fields] OR "tele intensive care"[All Fields] OR "tele intensive care"[All Fields] OR "tele icu"[All Fields] OR "tele icu"[All Fields] OR "Mobile Health"[All Fields] OR "mHealth"[All Fields] OR "Telehealth"[All Fields] OR "eHealth"[All Fields] OR "Internet based"[All Fields] OR "Online based"[All Fields] | [173,744](https://pubmed-ncbi-nlm-nih-gov-ssl.libproxy.snu.ac.kr/?term=%28%28%28%28Internet%5BMeSH+Terms%5D%29+OR+%28Internet+use%5BMeSH+Terms%5D%29%29+OR+%28Internet-based+intervention%5BMeSH+Terms%5D%29%29+OR+%28Telemedicine%5BMeSH+Terms%5D%29%29+OR+%28%22Internet+Uses%22+OR+%22Web+Usage%22+OR+%22Web+Use%22+OR+%22Internet+Usage%22+OR+%22Internet+Based+Intervention%22+OR+%22Internet-Based+Interventions%22+OR+%22Web-based+Intervention%22+OR+%22Web+based+Intervention%22+OR+%22Web-based+Interventions%22+OR+%22Online+Intervention%22+OR+%22Online+Interventions%22+OR+%22Internet+Intervention%22+OR+%22Internet+Interventions%22+OR+%22Tele-Referral%22+OR+%22Tele-Referrals%22+OR+%22Virtual+Medicine%22+OR+%22Tele-Intensive+Care%22+OR+%22Tele+Intensive+Care%22+OR+%22Tele-ICU%22+OR+%22Tele+ICU%22+OR+%22Mobile+Health%22+OR+%22mHealth%22+OR+%22Telehealth%22+OR+%22eHealth%22+OR+%22Internet+based%22+OR+%22Online+based%22+OR+%22Web+based%22%5BMeSH+Terms%5D%29&sort=) |
| #3 | "Quality of life"[MeSH Terms] OR "Life Quality"[All Fields] OR "health related quality of life"[All Fields] OR "health related quality of life"[All Fields] OR "HRQOL"[All Fields] OR "Psychological Well-Being"[All Fields] OR "QOL"[All Fields] OR "Well-Being"[All Fields] | [408,507](https://pubmed-ncbi-nlm-nih-gov-ssl.libproxy.snu.ac.kr/?term=%28quality+of+life%5BMeSH+Terms%5D%29+OR+%28%22Life+Quality%22+OR+%22Health-Related+Quality+Of+Life%22+OR+%22Health+Related+Quality+Of+Life%22+OR+%22HRQOL%22+OR+%22Psychological+Well-Being%22+OR+%22QOL%22+OR+%22Well-Being%22%29&sort=) |
| #4 | "Randomized Controlled Trial"[All Fields] OR "Randomised Controlled Trial"[All Fields] OR "Randomized"[All Fields] OR "Randomised"[All Fields] OR "Randomization"[All Fields] OR "Randomisation"[All Fields] | [1,098,702](https://pubmed-ncbi-nlm-nih-gov-ssl.libproxy.snu.ac.kr/?term=%22Randomized+Controlled+Trial%22%5BAll+Fields%5D+OR+%22Randomised+Controlled+Trial%22%5BAll+Fields%5D+OR+%22Randomized%22%5BAll+Fields%5D+OR+%22Randomised%22%5BAll+Fields%5D+OR+%22Randomization%22%5BAll+Fields%5D+OR+%22Randomisation%22%5BAll+Fields%5D&sort=) |
| #5 | #1 AND #2 AND #3 AND #4 | 148 |

**Supplementary Table 2. Web of Science**

| Search # | MeSH Terms and Key Words | Articles Revealed |
| --- | --- | --- |
| #1 | **ALL=("Breast Neoplasms" OR "Breast Neoplasm" OR "Breast Tumors" OR "Breast Tumor" OR "Breast Cancer" OR "Mammary Cancer" OR "Mammary Cancers" OR "Malignant Neoplasm of Breast" OR "Breast Malignant Neoplasm" OR "Breast Malignant Neoplasms" OR "Malignant Tumor of Breast" OR "Breast Malignant Tumor" OR "Breast Malignant Tumors" OR "Cancer of Breast" OR "Cancer of the Breast" OR "Human Mammary Carcinomas" OR "Human Mammary Carcinoma" OR “Human Mammary Neoplasms" )** | 624,246 |
| #2 | **ALL=("Internet Uses" OR "Web Usage" OR "Web Use" OR "Internet Usage" OR "Internet Based Intervention" OR "Internet-Based Intervention" OR "Internet-Based Interventions" OR "Web-based Intervention" OR "Web based Intervention" OR "Web-based Interventions" OR "Online Intervention" OR "Online Interventions" OR "Internet Intervention" OR "Internet Interventions" OR "Tele-Referral" OR "Tele-Referrals" OR "Virtual Medicine" OR "Tele-Intensive Care" OR "Tele Intensive Care" OR "Tele-ICU" OR "Tele ICU" OR "Mobile Health" OR "mHealth" OR "Telehealth" OR "eHealth" OR "Internet based" OR "Online based" OR "Web based" OR “Internet Access” OR “Internet Use” OR “Telemedicine”)** | 177,118 |
| #3 | **ALL=("Life Quality" OR "Health-Related Quality Of Life" OR "Health Related Quality Of Life" OR "HRQOL" OR "Psychological Well-Being" OR "QOL" OR “Well-Being” OR “Quality of life”)** | 709,544 |
| #4 | **ALL=("Randomized Controlled Trial" OR "Randomised Controlled Trial" OR "Randomized" OR "Randomised" OR "Randomization" OR "Randomisation")** | 1,080,750 |
| #5 | **#4 AND #3 AND #2 AND #1** | [296](https://www-webofscience-com-ssl.libproxy.snu.ac.kr/wos/woscc/summary/784b6e7b-e8f0-42b3-b896-ee937cb22dde-aa0f9b7a/relevance/1) |

**Supplementary Table 3. Cochrane Library**

| Search # | MeSH Terms and Key Words | Articles Revealed |
| --- | --- | --- |
| #1 | MeSH descriptor: [Breast Neoplasms] explode all trees OR "Breast Neoplasm" OR "Neoplasm, Breast" OR "Breast Tumors" OR "Breast Tumor" OR "Tumor, Breast" OR "Tumors, Breast" OR "Neoplasms, Breast" OR "Breast Cancer" OR "Cancer, Breast" OR "Mammary Cancer" OR "Cancer, Mammary" OR "Cancers, Mammary" OR "Mammary Cancers" OR "Malignant Neoplasm of Breast" OR "Breast Malignant Neoplasm" OR "Breast Malignant Neoplasms" OR "Malignant Tumor of Breast" OR "Breast Malignant Tumor" OR "Breast Malignant Tumors" OR "Cancer of Breast" OR "Cancer of the Breast" OR "Mammary Carcinoma, Human" OR "Carcinoma, Human Mammary" OR "Carcinomas, Human Mammary" OR "Human Mammary Carcinomas" OR "Mammary Carcinomas, Human" OR "Human Mammary Carcinoma" OR "Mammary Neoplasms, Human" OR "Human Mammary Neoplasm" OR "Human Mammary Neoplasms" OR "Neoplasm, Human Mammary" OR "Neoplasms, Human Mammary" OR "Mammary Neoplasm, Human" OR "Breast Carcinoma" OR "Breast Carcinomas" OR "Carcinoma, Breast" OR "Carcinomas, Breast" | 44,232 |
| #2 | MeSH descriptor: [Internet] explode all trees OR MeSH descriptor: [Internet Use] explode all trees OR MeSH descriptor: [Internet Use] explode all trees OR MeSH descriptor: [Telemedicine] explode all trees OR "Internet Uses" OR "Web Usage" OR "Web Use" OR "Internet Usage" OR "Internet Based Intervention" OR "Internet-Based Intervention" OR "Internet-Based Interventions" OR "Web-based Intervention" OR "Web based Intervention" OR "Web-based Interventions" OR "Online Intervention" OR "Online Interventions" OR "Internet Intervention" OR "Internet Interventions" OR "Tele-Referral" OR "Tele-Referrals" OR "Virtual Medicine" OR "Tele-Intensive Care" OR "Tele Intensive Care" OR "Tele-ICU" OR "Tele ICU" OR "Mobile Health" OR "mHealth" OR "Telehealth" OR "eHealth" OR "Internet based" OR "Online based" OR "Web based" | 28,322 |
| #3 | MeSH descriptor: [Quality of Life] explode all trees OR "Life Quality" OR "Health-Related Quality Of Life" OR "Health Related Quality Of Life" OR "HRQOL" OR "Psychological Well-Being" OR "QOL" OR “Well-Being” | 85,048 |
| #4 | MeSH descriptor: [Randomized Controlled Trial] explode all trees OR Randomized Controlled Trial OR Randomized OR Randomised OR Randomization OR Randomisation | 1,211,049 |
| #5 | #1 AND #2 AND #3 AND #4 | 214 |

**Supplementary Table 4. CINAHL**

| Search # | Subject Headings (MH) and Key Words | Articles Revealed |
| --- | --- | --- |
| S1 | (MH "Breast Neoplasms") OR TX ( "Breast Neoplasm" OR "Breast Tumors" OR "Breast Tumor" OR "Breast Cancer" OR "Mammary Cancer" OR "Mammary Cancers" OR "Malignant Neoplasm of Breast" OR "Breast Malignant Neoplasm" OR "Breast Malignant Neoplasms" OR "Malignant Tumor of Breast" OR "Breast Malignant Tumor" OR "Breast Malignant Tumors" OR "Cancer of Breast" OR "Cancer of the Breast" OR "Human Mammary Carcinomas" OR "Human Mammary Carcinoma" OR “Human Mammary Neoplasms" ) | **158,117** |
| S2 | (MH "Internet")  OR (MH "Internet-Based Intervention") OR (MH "Telemedicine") OR TX ( "Internet Uses" OR "Web Usage" OR "Web Use" OR "Internet Usage" OR "Internet Based Intervention" OR "Internet-Based Intervention" OR "Internet-Based Interventions" OR "Web-based Intervention" OR "Web based Intervention" OR "Web-based Interventions" OR "Online Intervention" OR "Online Interventions" OR "Internet Intervention" OR "Internet Interventions" OR "Tele-Referral" OR "Tele-Referrals" OR "Virtual Medicine" OR "Tele-Intensive Care" OR "Tele Intensive Care" OR "Tele-ICU" OR "Tele ICU" OR "Mobile Health" OR "mHealth" OR "Telehealth" OR "eHealth" OR "Internet based" OR "Online based" OR "Web based" OR “Internet Access” ) | 145,833 |
| S3 | (MH "Quality of Life") OR TX ("Life Quality" OR "Health-Related Quality Of Life" OR "Health Related Quality Of Life" OR "HRQOL" OR "Psychological Well-Being" OR "QOL" OR “Well-Being” ) | 317,749 |
| S4 | PT randomized controlled trials OR TX ( "Randomized Controlled Trial" OR "Randomised Controlled Trial" OR "Randomized" OR "Randomised" OR "Randomization" OR "Randomisation" ) | 499,428 |
| S5 | S1 AND S2 AND S3 AND S4 (Limiters -Human) | 126 |

**Supplementary Table 5. Embase**

| Search # | Emtree Terms and Key Words | Articles Revealed |
| --- | --- | --- |
| #1 | 'breast cancer'/exp OR 'breast neoplasm' OR 'breast tumors' OR 'breast tumor' OR 'breast cancer' OR 'mammary cancer' OR 'mammary cancers' OR 'malignant neoplasm of breast' OR 'breast malignant neoplasm' OR 'breast malignant neoplasms' OR 'malignant tumor of breast' OR 'breast malignant tumor' OR 'breast malignant tumors' OR 'cancer of breast' OR 'cancer of the breast' OR 'human mammary carcinomas' OR 'human mammary carcinoma' OR 'human mammary neoplasms' | 750,294 |
| #2 | 'internet'/exp OR 'internet use'/exp OR 'web-based intervention'/exp OR 'web-based intervention'/exp OR 'internet uses' OR 'web usage' OR 'web use' OR 'internet usage' OR 'internet based intervention' OR 'internet-based intervention' OR 'internet-based interventions' OR 'web-based intervention' OR 'web based intervention' OR 'web-based interventions' OR 'online intervention' OR 'online interventions' OR 'internet intervention' OR 'internet interventions' OR 'tele-referral' OR 'tele-referrals' OR 'virtual medicine' OR 'tele-intensive care' OR 'tele intensive care' OR 'tele-icu' OR 'tele icu' OR 'mobile health' OR 'mhealth' OR 'telehealth' OR 'ehealth' OR 'internet based' OR 'online based' OR 'web based' OR 'internet access' | 238,685 |
| #3 | 'quality of life'/exp OR 'life quality' OR 'health-related quality of life' OR 'health related quality of life' OR 'hrqol' OR 'psychological well-being' OR 'qol' OR 'well-being' | 819,267 |
| #4 | 'randomized controlled trial'/exp OR 'randomized controlled trials' OR randomized OR randomised OR randomization OR randomisation | 1,558,008 |
| #5 | #1 and #2 and #3 and #4 | 348 |

**Supplementary Table 6.** [**PsycINFO**](https://lib.snu.ac.kr/find/dblink/?dbid=340)

| Search # | Main Subjects and Key Words | Articles Revealed |
| --- | --- | --- |
| S1 | DE "Breast Neoplasms" OR TX ( "Breast Neoplasm" OR "Breast Tumors" OR "Breast Tumor" OR "Breast Cancer" OR "Mammary Cancer" OR "Mammary Cancers" OR "Malignant Neoplasm of Breast" OR "Breast Malignant Neoplasm" OR "Breast Malignant Neoplasms" OR "Malignant Tumor of Breast" OR "Breast Malignant Tumor" OR "Breast Malignant Tumors" OR "Cancer of Breast" OR "Cancer of the Breast" OR "Human Mammary Carcinomas" OR "Human Mammary Carcinoma" OR “Human Mammary Neoplasms ) | 16,202 |
| S2 | ( DE "Internet" OR DE "Internet Access" OR DE "Internet Usage" OR DE "Digital Interventions" OR DE “Telemedicine" ) OR TX ( "Internet Uses" OR "Web Usage" OR "Web Use" OR "Internet Usage" OR "Internet Based Intervention" OR "Internet-Based Intervention" OR "Internet-Based Interventions" OR "Web-based Intervention" OR "Web based Intervention" OR "Web-based Interventions" OR "Online Intervention" OR "Online Interventions" OR "Internet Intervention" OR "Internet Interventions" OR "Tele-Referral" OR "Tele-Referrals" OR "Virtual Medicine" OR "Tele-Intensive Care" OR "Tele Intensive Care" OR "Tele-ICU" OR "Tele ICU" OR "Mobile Health" OR "mHealth" OR "Telehealth" OR "eHealth" OR "Internet based" OR "Online based" OR "Web based" OR “Internet Access” OR “Internet Use” ) | 72,601 |
| S3 | ( DE "Quality of Life" OR DE "Health Related Quality of Life" OR DE "Well Being" ) OR TX ( "Life Quality" OR "Health-Related Quality Of Life" OR "HRQOL" OR "Psychological Well-Being" OR "QOL" ) | 136,398 |
| S4 | ( DE "Randomized Controlled Trials" OR DE "Randomized Clinical Trials" ) OR TX ( "Randomized Controlled Trial" OR "Randomised Controlled Trial" OR "Randomized" OR "Randomised" OR "Randomization" OR "Randomisation" | 113,918 |
| S5 | S1 AND S2 AND S3 AND S4 | 45 |
